# Profiling of the Predicted Circular RNAs in Ductal In Situ and Invasive Breast Cancer: A Pilot Study

**DOI:** 10.1155/2016/4503840

**Published:** 2016-11-14

**Authors:** Marco Galasso, Giorgio Costantino, Lorenzo Pasquali, Linda Minotti, Federica Baldassari, Fabio Corrà, Chiara Agnoletto, Stefano Volinia

**Affiliations:** ^1^LTTA, Department of Morphology, Surgery and Experimental Medicine, University of Ferrara, Via Fossato di Mortara 70, 44123 Ferrara, Italy; ^2^Department of Medicine, Center for Molecular Medicine (CMM) L8:02, Karolinska Institutet, 17176 Stockholm, Sweden

## Abstract

The recent advantage obtained by next generation sequencing allows a depth investigation of a new “old” kind of noncoding transcript, the circular RNAs. Circular RNAs are nontranslated RNAs, typically nonpolyadenylated, with a resistance to exonucleases that gives them the ability to be more stable than the common linear RNA isoforms. We used a bioinformatic detection tool (CIRCexplorer) to research predictive circRNAs from the next generation sequenced data of five samples of ductal in situ carcinoma (DCIS) and matched adjacent invasive ductal carcinoma (IDC). Furthermore, we also investigated the circular RNAs expressed in MCF7, an invasive breast ductal carcinoma cell line. We described the genomic context of the predicted circular RNAs and we address the hypothetical possible functional roles. This study showed a perspective of a panel of predictive circRNAs identified and the function that circRNAs could exert.

## 1. Introduction

Annually more than a million new diagnoses worldwide for breast cancer (BC) emerge, becoming the most common female malignancy [1]. The term “breast cancer” refers to a complex disease, displaying distinctive histologic and genetic features. Dramatic changes occur in gene expression during the transformation of normal breast tissues into ductal in situ carcinoma (DCIS), much less important, and somewhat surprisingly, in the transition from DCIS to invasive ductal carcinoma (IDC). In fact, those from the same histological subtype share very similar genetic, epigenetic alterations and gene expression patterns. DCIS and matched adjacent IDC (synchronous DCIS and IDC) have remarkably similar genetic and copy number profile [2, 3]. Therefore, none of the current biomarkers, or genomic classifications, can predict if, and when, DCIS will progress to IDC. In principle, a better understanding of DCIS progression could highlight ways to arrest invasion at the preinvasive stage [4]. From coding to noncoding, high-throughput next generation sequencing (NGS) has been analyzing in depth the entire genome at base-pair resolution. The noncoding panorama is a real opportunity to reveal new biomarkers or potential targets: in this landscape long noncoding RNAs (lncRNAs) are an emerging class of key regulatory RNAs that do not code for protein and are not translated [5]. lncRNAs are crucial players in various key biological processes that include dosage compensation, genomic imprinting, chromatin organization, gene regulation, and alternative splicing [6]. lncRNA mechanism of action need still to be widely revealed [7]. More recently, the research interest is focused on circular RNAs (circRNAs), a large class of long noncoding RNAs circularized and discovered from a handful of transcribed genes more than twenty years ago [8]. Nowadays, there are different types of circRNAs [9] and are known to be abundant, conserved, and stable in cytoplasm and even in blood. At the time of the discovery these RNA molecules had generally been considered to be of low abundance and likely representing errors in splicing. CircRNAs are nontranslated RNAs, typically nonpolyadenylated, with a resistance to exonucleases that gives them the ability to be more stable than the common linear RNA isoforms, also increasing proper folding by imposing structural constraints on the RNA [10]. Their formation is due to a non-random back-splice event that involves in a covalent junction between the 3′ and 5′ ends, providing covalently closed continuous loops configuration, in opposition to the theory that for decades considering the circRNAs as the result of gene rearrangements or splicing artifacts producing rare isoforms [10, 11]. Several studies revealed that circRNAs are usually composed of 1 to 5 exons, where each exon can be up to three times longer than the average expressed exon. This perhaps suggests that the probability to circularizing for an exon is directly proportional to the length of that exon [12]. Although the formation mechanism and the cellular function have been completely understood, the function and the expressions are not clear. The majority of circRNAs often exhibit tissue/developmental-stage-specific expression, described recently for the brain [13]. CircRNAs are also deregulated in cancer cell lines [11], but not many studies have been performed yet on cancer patients. Recently, some studies have been providing evidences about the interaction between circRNA and miRNA, supporting a “sponge effect” of the circRNAs as posttranscriptional regulators of other ncRNAs, such as miRNAs [10, 14]. Based on these findings, we will define the panel of expressed circRNAs, in DCIS and IDC samples. We predicted strong circRNAs candidates, enforcing their existence with an independent false discovery polyadenylated subtraction, and then we studied the possible roles of selected circRNAs.

## 2. Material and Methods

### 2.1. Data

Publicly available RNA-seq data of total RNA from ten tandem DCIS/IDC samples from five patients afflicted with DCIS that are synchronous with IDC within the same breast (GSE66301, downloaded from the Sequence Read Archive (SRA)) (Illumina GAII, paired-end sequencing (2 × 75 nt length)). GSE66301 series reports data from 6 pairs of DCIS-IDC samples, we excluded one pair that has one sample that not respected the high media level (>88%) of the mapping quality reads percentage, showing the 49%. Reference genome human hg19 (February 2009, GRCh37), was downloaded from the UCSC genome browser (http://genome.ucsc.edu/). For CIRCexplorer we used STAR as aligner software [15]. We also used the fastq.gz samples of the MCF7 cell line. These are public and available at the ENCODE repository site (http://hgdownload.cse.ucsc.edu/goldenpath/hg19/encodeDCC/): poly(A) and nonpoly(A) selected RNA-seq data, generated with paired-end sequencing (2 × 75 nt length), after the trimming quality control steps.

### 2.2. Data Quantification and Statistical Analysis

For circRNAs, each junction required supported from at least two independent reads within the sample. The number of reads for each circRNAs identified was used as measure of expression. The raw read counts were normalized to sequencing depth by dividing the raw number with the number of total reads mapped to and multiply for 10^9^ (SRPBM: reads per billion mapped reads). RPM (reads per million) was used for the mRNAs. The circBase track on genome browser was consulted to verify the annotation and if the circRNAs was showed by other previous experiments [16]. For the enrichment analysis of the potential disease involved with expression of circRNAs we used the Circ2Trait tool [17]. miRTarbase (http://miRTarBase.mbc.nctu.edu.tw/) was used to find the genes involved in EMT and targeted by the human miR-200b, miR-200c, and miR.429. Custom pipelines to identify: different and common circRNAs, and to create graphics, were performed using R software (http://www.R-project.org/).

### 2.3. Bioinformatic Pipeline

The raw total RNA sequencing data, after quality control procedures (trimming), was used for running the bioinformatic detection pipeline: CIRCexplorer. We used this algorithm because it was one of the mostly used and cited in many sounded scientific research; and in comparison to other four pipelines, it has shown high accuracy and a good sensitivity [18]. Briefly, CIRCexplorer was focused on the identification of junction reads from back-spliced exons and intron lariats (the terminal parts of all unmapped reads that are aligned independently to the genome) [19]. In parallel, we analyzed circular RNAs expression of the total RNA-seq of MCF7, an invasive breast ductal carcinoma cell line. This analysis revealed us the candidates circular RNAs expressed in polyadenylated (polyA+) and not polyA+ selected samples. Previous studies have reported that circRNAs were nonpolyadenylated [11] or RNase R-resistant [12], then we expected that the circRNAs discovered using poly(A)+ selection could be the results of miscellaneous amplification products, misalignment on references genomes or false positives, probably not well filtered by the quality control software. All the circRNAs, predicted in the polyA+ samples, were considered false positive and then used as criteria of exclusion. Basically, we applied a false discovery polyadenylated subtraction from the output lists of all the circRNAs candidates of each samples. Moreover, the circRNAs were included by at least two supporting reads. Following these selection criteria this procedure would gave us a list of high-confidence circRNAs. Here we listed an example of the command parameters used for “CIRCexplorer pipeline”:* //Alignment* STAR -* *-genomeDir “hg19_index” -* *-readFilesIn “fastq1” “fastq2” -* *-readFilesCommand zcat -* *-runThreadN 8 -* *-chimSegmentMin 20 -* *-chimJunctionOverhangMin 20 -* *-outFileNamePrefix $STARout_dir 1≫“log_file” 2≫“err_file”* //convert chimeric to file.txt.*/star_parse.py Chimeric.out.junction fusion_junction.txt* //search circRNAs.*/CIRCexplorer.py -j fusion_junction.txt -g “GENOME.fa” -r “ANNOTATION.txt”.

## 3. Results and Discussion

### 3.1. The Genomic Context of the Predicted Circular RNAs in Breast Cancer Samples

**Figure 1:**
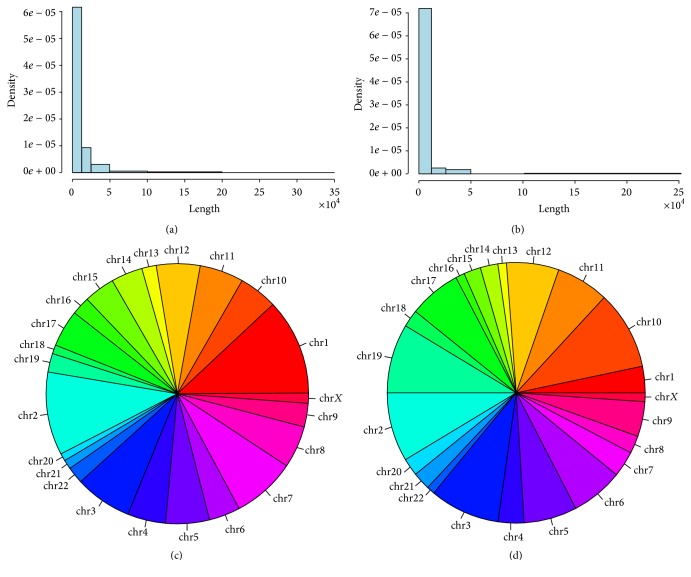
The histograms showed the presence of predicted circRNAs (*y-axis*) related to their length (*x-axis*) in the totality of DCIS (a) and IDC (b) samples. The pie chart was divided into slices to illustrate numerical proportion of circRNAs predicted for each chromosome in the totality of DCIS (c) and IDC (d) samples.

DCIS invasive progression has been characterized by investigating the circRNAs expression in tandem DCIS/IDC model using five samples patients afflicted with DCIS that were synchronous with IDC within the same breast (GSE66301) [20]. We obtained for each sample the list of circRNAs candidates from algorithms' output and refined by the false positive subtraction (see Section 2 for details). As expected, there were differences in terms of the total amount of circRNAs identified in each sample: the CIRCexplorer found 1938 raw circRNAs with one read. The total candidate distinct circular RNAs that passed our criteria were 756 in the total of the breast cancer: 552 expressed in DCIS and 208 in IDC (Supplementary Table 1 in Supplementary Material available online at http://dx.doi.org/10.1155/2016/4503840). Characterizing the genomic context of the predicted circRNAs, we firstly described the genomic localization of this class of noncoding RNAs. In DCIS the 80% (443/552) of circular RNAs belonged to the “exonic circRNAs” class derived from back-spliced and the 20% belonged to ciRNAs (circular intronic RNA). ciRNAs were a form of intron lariats that have been circularized at the branchpoint 2′–5′ linkage; normally degraded from the 3′ end up to the branchpoint, ciRNAs were stabilized in some way not yet clearly described. IDC showed a different profile: 55% (112/204) were ciRNAs, the remaining were “exonic circRNAs”. The majority of the circular RNAs identified had a length under the 12500 nucleotides in both the classes studied (Figures 1(a) and 1(b) and Supplementary Figure 1). The circRNAs production can involve more than one exon, we found up to 16 exons involved both in DCIS and IDC. As expected, there was a significant positive Spearman's rank correlation between the length and the number of exons involved in the circularization in DCIS (rho = 0.844, 1 tail *p* value < 2.2*e *− 16) and IDC too (rho = 0.630, 1 tail *p* value < 2.2*e *− 16). The chromosomes (chr) most represented in terms of circRNAs were showed in Figures 1(c) and 1(d): chr1, chr2, and chr3 in DCIS, while in IDC there were chr2, chr3, chr10, and chr19.

### 3.2. Common and Different circRNAs Were Expressed in Synchronous DCIS and IDC Samples

**Figure 2:**
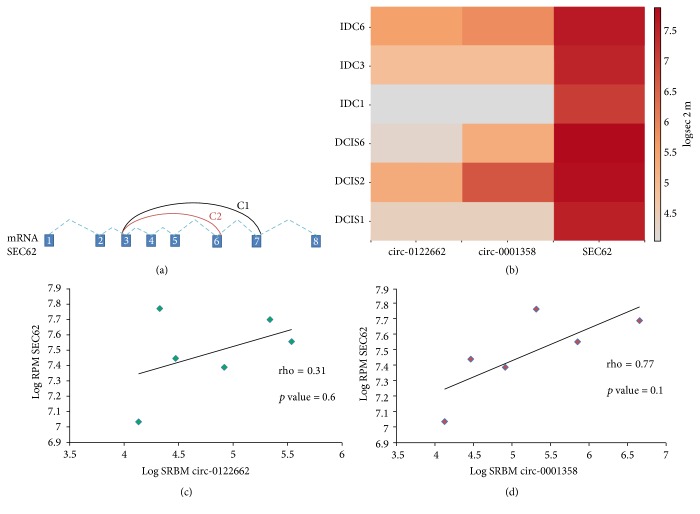
Schematic illustration of the two circRNAs C1 (hsa-circ-0001358) and C2 (hsa-circ-0122662) produced by the pre-mRNA of SEC62 gene. The heatmap showed the log expression values of the hsa-circ-0122662, hsa-circ-001803, and SEC62 in the two paired samples and two unpaired (b). The scatterplot showed the correlation between SEC62 (*y-axes*) and the two circular RNAs, respectively (*x-axes*): hsa-circ-0122662 and hsa-circ-0001358. Rho and *p* values were obtained performing a Spearman rank test (c and d).

Two circular RNAs, belonging to two different genes, FBXW4 and PSMA7, were found in common between the IDC samples and MCF7 cell line. These data did not reflect the gene expression profiled studies and they may suggest that these discrepancies could be due to the different technology used, or that circRNAs would be mostly related to the environment where the cancer cells were growing. A panel of 18 circRNAs were both expressed in at least one DCIS and IDC sample (Table 1 and Supplementary Figure 2). Only one coding gene, SEC62 (also named TLOC1), showed two circular RNAs that differed in terms of number of the exons involved. These two circRNAs were located in the chromosome region 3q26.2, that it is interestingly frequent amplified in several cancers [21]. The longest in terms of nucleotides (11414 nts) was the hsa-circ-0001358 (alias hsa-circ-001803), while the second was hsa-circ-0122662, 8920 nts long. There were no significant differences in terms of expression between these two circRNAs comparing the levels of expression of DCIS and IDC classes (Wilcoxon-Mann-Whitney, *p* value = 0.8). The SEC62 mRNA was expressed more than the circular RNAs isoforms (Figure 2(b)) and the biological trend of expression suggested a positive correlation between the mRNA and the two circular RNAs (Figures 2(c) and 2(d)), but this hypothesis was not significantly confirmed by the Spearman rank test. To figure out the possible involvements of these circRNAs as “sponges” for the miRNAs, we investigated if there were possible target binding sites in their sequences.  Table 2 listed five possible miRNAs that can interact with the hsa-circ-0001358, no interactions were found for the hsa-circ-0122662. The miRNAs interacting with the circRNA were obtained using the Starbase human Pan cancer tool [22]. The alignment scores of each miRNAs were showed in Supplementary Figure 3. Of note, a decreased expression of MicroRNA-200 family (in Table 2 were listed three members: miR-200b-3p, miR-200c-3p, and miR-429-3p) was associated to migration and invasion of breast cancer cells in vitro in several reports in literature [23, 24]. Therefore, we investigated the most important genes involved in Epithelial Mesenchymal Transitions (EMT) and that were targeted by the microRNA-200 family to figure out a putative circRNA/miRNA/mRNA axis involved in DCIS to IDC progression (Supplementary Table 2). No significant correlations were found investigating the hsa-circ-0001358 and the ZEB1 (Zinc Finger E-Box Binding Homeobox 1), ZEB2 (Zinc Finger E-Box Binding Homeobox 2), and VIM (Vimentin) mRNAs levels. Intriguingly, two targets of the miRNAs showed an opposite behavior: BMI-1 (B lymphoma Mo-MLV insertion region 1 homolog) positively correlated (rho = 0.886 and *p* values = 0.02) with the circ-0001358, instead FN1 (Fibronectin 1) showed a negative correlation (rho = 0.886 and *p* values = 0.02). Lastly, using circ2traits, we searched the measure of likelihood of the hsa-circ-0001358, to figure out possible association with the 174 disease reported in the circ2traits database. Breast cancer was the third on twenty-four significantly associated disease (*p* value = 0.002, Supplementary Table 3). Interestingly, we found many circRNAs expressed only in DCIS or IDC, that could represent a difference in terms of selected dynamic expression. This data will be in depth investigated for a better understanding of the DCIS progression to invasive.

## 4. Conclusion

We have described a panel of predicted circRNAs investigating a pilot small cohort of samples having DCIS and IDC synchronous. We have to take into account two limitations of this study: (i) since the majority of circRNAs were expressed at very low levels, and we analyzed total RNA sequencing data without the use of RNase R, an enzyme that digests linear RNAs but preserves circRNAs [25], the number of circRNAs identified was lower respect the media of other reports in literature. Therefore only an RNase R treatment will permit to enrich circRNAs and then to further verify the existence of the predicted circular RNAs. (ii) The second weakness point was the small size of this cohort. To further address the potential effect of the predicted circRNAs, it will be fundamental enlarge the cohort and perform molecular biological experiments that might permit to validate these new findings. Altogether these data try also to shed light on the possible involvement of a circRNA/miRNA/mRNA axis, but these data are still preliminary and in need of a further validation. In the next future, this class of noncoding transcripts, still largely uncharted, will be revealed important in biomedical studies, especially in those seeking to find useful marker for diagnosis and prognosis and maybe to develop new therapeutics targets.

## Supplementary Material

The Supplementary File was composed by three Supplementary Tables and the legends of the Supplementary Figures.
Supplementary Table 1 showed the numbers of circRNAs predicted.
Supplementary Table 2 listed Spearman correlation rho values for each of the EMT genes studied in the cohort.
Supplementary Table 3 listed the diseases enriched for the candidate circRNA using the circ2traits database.
Supplementary Figure 1 showed the plot of the density presence of predicted circRNAs.
Supplementary Figure 2 showed the heatmap of log values of 18 expressed circRNAs.
Supplementary Figure 3 showed the alignment of each miRNAs against the candidate circRNA.

## Figures and Tables

**Table 1:** The 18 circRNAs expressed in at least one DCIS and IDC sample were listed. The genomic information and the chromosomal coordinate were related to the reference genome human hg19 (GRCh37). Ensemble isoform transcript id names were reported.

Chromosomal location	circBASE number	Length	Strand	Exon count	Exon sizes	Gene name	Isoform name
chr2: 232325188–232325275	0001111, alias 001797	87	−	1	87	NCL	ENST00000322723.4
chr2: 99976698–99988193	NA	11495	+	8	126, 85, 673, 218, 151, 99, 90, 75	EIF5B	ENST00000289371.6
chr3: 169694733–169703653	0122662	8920	+	4	106, 205, 93, 61	SEC62	ENST00000337002.4
chr3: 169694733–169706147	0001358, alias 001803	11414	+	5	106, 205, 93, 61, 120	SEC62	ENST00000337002.4
chr5: 179146668–179146782	0075303	114	+	1	114	CANX	ENST00000504734.1
chr5: 81572220–81572269	NA	49	−	1	49	RPS23	ENST00000510019.1
chr6: 128718710–128718833	0077818	123	−	1	123	PTPRK	ENST00000368226.4
chr7: 11021998–11030474	0133015	8476	+	2	788, 145	PHF14	ENST00000403050.3
chr9: 5968018–5988545	0138872	20527	−	2	1, 418, 201	KIAA2026	ENST00000399933.3
chr10: 112360196–112360304	NA	108	+	1	108	SMC3	ENST00000361804.4
chr11: 64888971–64889011	NA	40	−	1	40	FAU	ENST00000525297.1
chr11: 70266505–70266616	0023341	111	+	1	111	CTTN	ENST00000346329.3
chr14: 105235723–105235829	NA	106	+	1	106	RP11-982M15.2	ENST00000557223.1
chr15: 42807434–42807552	NA	118	+	1	118	SNAP23	ENST00000249647.3
chr17: 48271709–48271808	NA	99	−	1	99	COL1A1	ENST00000225964.5
chr19: 50358226–50361371	0051970	3145	+	5	108, 156, 90, 74, 58	PTOV1	ENST00000600603.1
chr19: 9938619–9938687	NA	68	+	1	68	UBL5	ENST00000590068.1
chr20: 25252018–25252122	NA	104	+	1	104	PYGB	ENST00000216962.4

NA = not available.

**Table 2:** The table showed the five miRNAs predicted to interact with SEC62 and hsa-circ-0001358. The clip read numbers were the total reads found in ClipSeq experiments. The bioComplex column represented the numbers of the high-throughput sequencing of RNA isolated by crosslinking immunoprecipitation (AGO2 HITS-CLIP) of *n* cell lines.

Name	mirAccession	TargetSites	bioComplex	ClipReadNum	TargetLocation
hsa-miR-200c-3p	MIMAT0000617	1	3	60	chr3: 169706110–169706132
hsa-miR-200b-3p	MIMAT0000318	1	3	60	chr3: 169706111–169706132
hsa-miR-429	MIMAT0001536	1	3	60	chr3: 169706112–169706132
hsa-miR-376a-3p	MIMAT0000729	1	8	76	chr3: 169701040–169703613
hsa-miR-376b-3p	MIMAT0002172	1	8	76	chr3: 169701039–169703613
