# Supplementary material for: Profiling of the Predicted Circular RNAs in Ductal In Situ and Invasive Breast Cancer: A Pilot Study
Source: Int J Genomics. 2016 Nov 14;2016:4503840. doi: 10.1155/2016/4503840 (PMC5124670; doi:10.1155/2016/4503840)

Supplementary Files

Supplementary Table 1. The numbers of circRNAs predicted before and after the filtering criteria described in Material and Methods. The final percentage of predicted circRNAs that pass the filtering procedure is reported.

	DCIS1	DCIS2	DCIS3	DCIS4	DCIS6	IDC1	IDC2	IDC3	IDC4	IDC6
**Number of circRNAs pre filtering procedure**	**243**	**188**	**68**	**88**	**916**	**84**	**85**	**94**	**57**	**115**
**Number of circRNAs after filtering procedure**	**81**	**61**	**41**	**25**	**360**	**57**	**17**	**27**	**37**	**70**
Percentage	**33.33**	**32.45**	**60.29**	**28.41**	**39.30**	**67.86**	**20.00**	**28.72**	**64.91**	**60.87**

Supplementary Table 2. Spearman correlation rho values were listed for each of the EMT genes studied in the cohort. VIM and CDH1 were not targeted by miRNAs investigated, but were included because are important EMT markers.

Genes	miRTarBase	Spearman rho	2 tails p-values
**ZEB1**	miR-200c - miR200b - miR-429	-0.3142857	0.5441
**ZEB2**	miR-200c - miR200b - miR-429	-0.08571429	0.8717
**BMI**	miR-200c - miR200b	**0.8857143**	**0.01885**
**FN1**	miR-200c - miR200b	**-0.8857143**	**0.01885**
**VIM**	-	-0.4285714	0.3965
**CDH1**	-	0.7714286	0.0724

Supplementary Table 3. The table listed the diseases enriched for the hsa-circ-001803 using the circ2traits database.

**ID circRNA**	**Disease**	***p-values***
hsa_circ_001803	lung cancer	1.16E-10
hsa_circ_001803	non-small cell lung cancer	3.01E-06
**hsa_circ_001803**	**breast cancer**	**0.001762331**
hsa_circ_001803	head and neck squamous cell carcinoma	0.001762331
hsa_circ_001803	ovarian cancer	0.001762331
hsa_circ_001803	adenoma	0.029454035
hsa_circ_001803	bladder cancer	0.029454035
hsa_circ_001803	Burkitt lymphoma	0.029454035
hsa_circ_001803	cholangiocarcinoma	0.029454035
hsa_circ_001803	colorectal cancer	0.029454035
hsa_circ_001803	coronary artery disease	0.029454035
hsa_circ_001803	gastric cancer	0.029454035
hsa_circ_001803	glioma	0.029454035
hsa_circ_001803	hamartoma	0.029454035
hsa_circ_001803	laryngeal carcinoma	0.029454035
hsa_circ_001803	lipoma	0.029454035
hsa_circ_001803	malignant melanoma	0.029454035
hsa_circ_001803	myoma	0.029454035
hsa_circ_001803	prostate cancer	0.029454035
hsa_circ_001803	sarcoma	0.029454035
hsa_circ_001803	squamous carcinoma	0.029454035
hsa_circ_001803	tongue squamous cell carcinoma	0.029454035
hsa_circ_001803	uterine leiomyoma	0.029454035

Supplementary Figures

Supplementary Figure 1. The plot showed the density presence of predicted circRNAs (*y axes*) related to their length (x *axes*) comparing the DCIS (rose) and IDC (blue) samples.

Supplementary Figure 2. The heatmap showed the log expression values of 18 predicted circRNAs *(y axis)* . The name of each circRNAs was reported including the name of the genes the circRNAs derived from. All the samples were listed *(x-axis).* Log2 SRBM values were reported. Black label showed no detectable levels.

Supplementary Figure 3. The alignment scores of each miRNAs against hsa-circ-0001358 were reported. The alignments were obtained using the Starbase human Pan cancer tool.

Supplementary Figure 1.

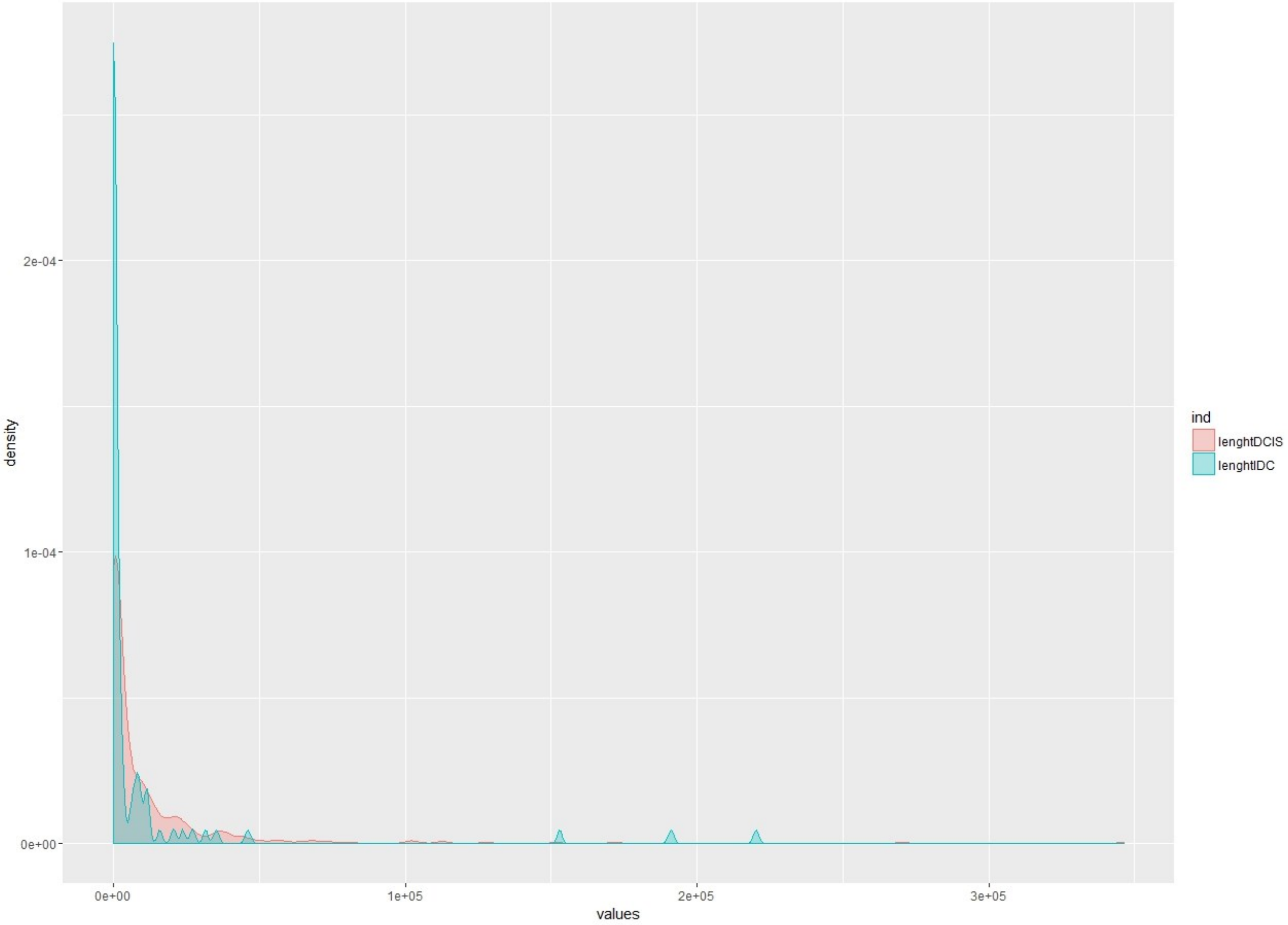

Supplementary Figure 2.

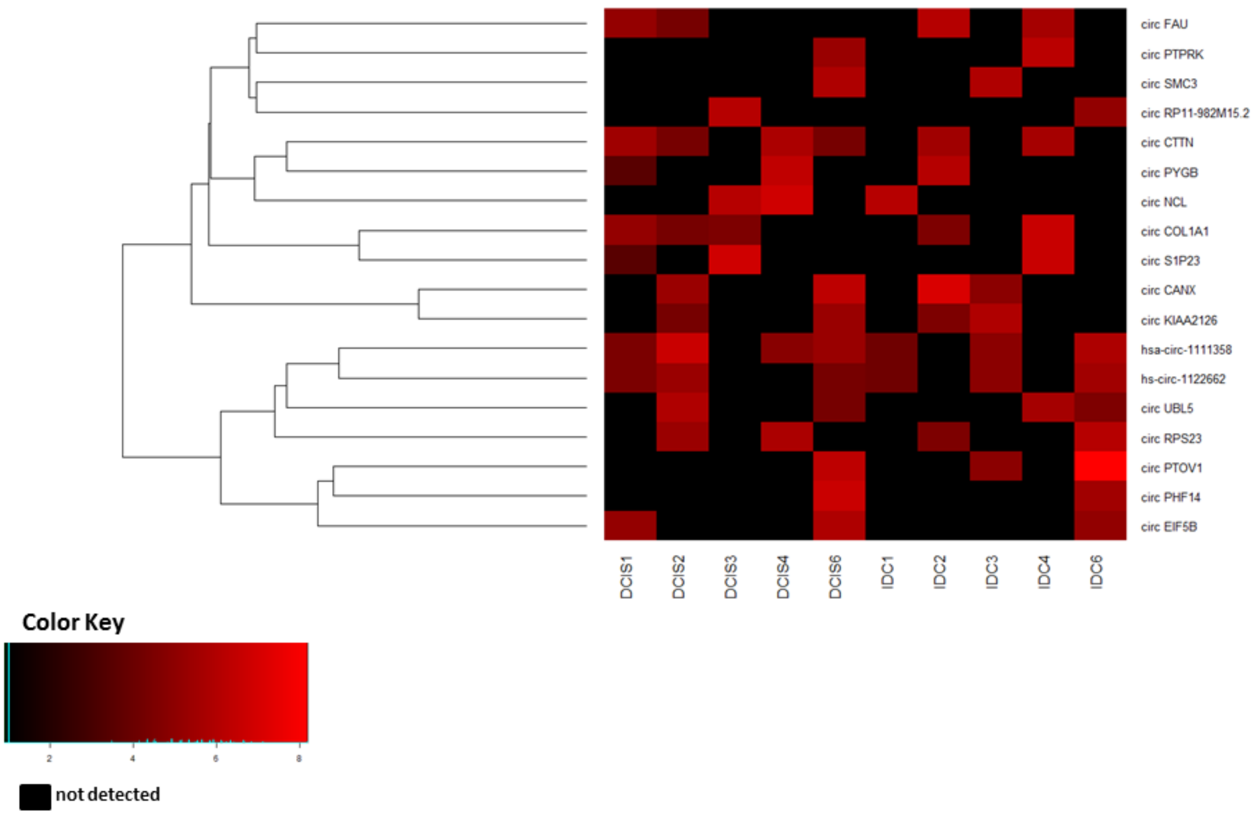

Supplementary Figure 3.

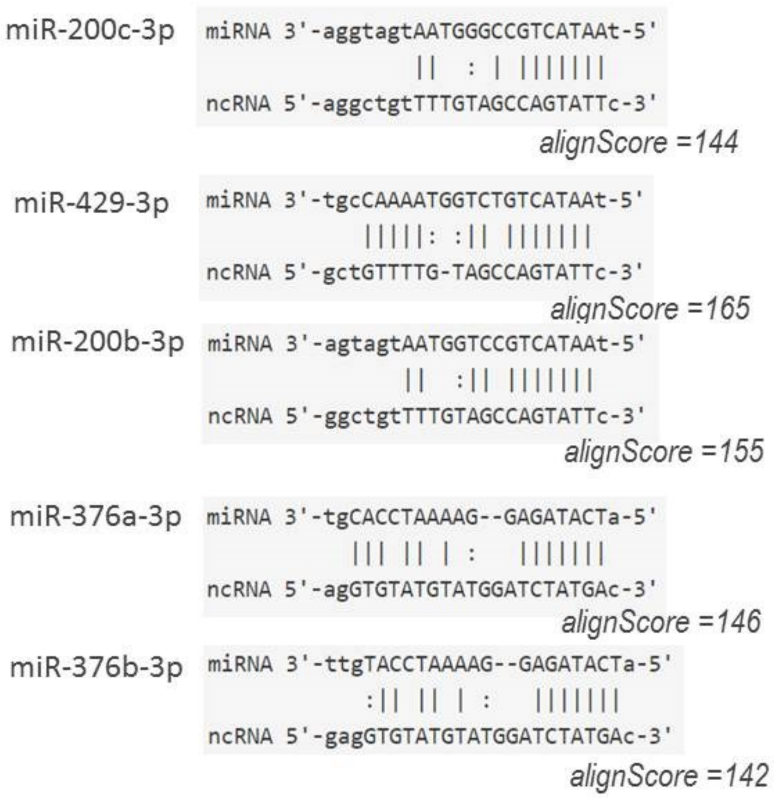

Supplement: Supplementary file 1 — The Supplementary File was composed by three Supplementary Tables and the legends of the Supplementary Figures. Supplementary Table 1 showed the numbers of circRNAs predicted. Supplementary Table 2 listed Spearman correlation rho values for each of the EMT genes studied in the cohort. Supplementary Table 3 listed the diseases enriched for the candidate circRNA using the circ2traits database. Supplementary Figure 1 showed the plot of the density presence of predicted circRNAs. Supplementary Figure 2 showed the heatmap of log values of 18 expressed circRNAs. Supplementary Figure 3 showed the alignment of each miRNAs against the candidate circRNA. [file 4503840.f1.pdf]
